# Reversibly Tuning the Viscosity of Peptide‐Based Solutions Using Visible Light

**DOI:** 10.1002/chem.202400544

**Published:** 2024-03-15

**Authors:** Simona Bianco, Laura Wimberger, Yael Ben‐Tal, George T. Williams, Andrew J. Smith, Jonathon E. Beves, Dave J. Adams

**Affiliations:** ^1^ School of Chemistry University of Glasgow Glasgow G12 8QQ UK; ^2^ School of Chemistry, UNSW Sydney Sydney NSW 2052 Australia; ^3^ School of Chemistry University of Southampton Southampton SO17 1BJ UK; ^4^ Institute for Life sciences University of Southampton Southampton SO17 1BJ UK; ^5^ Diamond Light Source Ltd., Diamond House Harwell Science and Innovation Campus Didcot Oxfordshire OX11 0DE UK

**Keywords:** light–triggered, reversible, Self–assembly, supramolecular, viscosity

## Abstract

Light can be used to design stimuli–responsive systems. We induce transient changes in the assembly of a low molecular weight gelator solution using a merocyanine photoacid. Through our approach, reversible viscosity changes can be achieved via irradiation, delivering systems where flow can be controlled non‐invasively on demand.

## Graphical abstract

A photo–switchable merocyanine photoacid has been used to induce reversible pH drops during the assembly of the modified dipeptide molecule 1ThNapFF. As the pH is decreased, protonation of 1ThNapFF leads to increased aggregation and increased viscosity. These reversible viscosity changes can be used to design systems where flow can be controlled in a non–invasive and localised way.

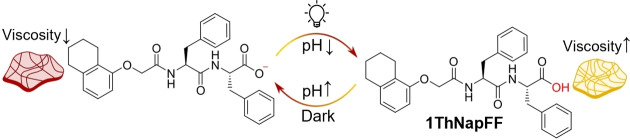

Driven by inspiration of biological systems that operate out–of–equilibrium, the design of systems capable of undergoing transient changes in self–assembly have gained significant interest in the field of systems chemistry.[^1^, ^2^, ^3^] Peptide–based low molecular weight gelators (LMWGs) are interesting candidates for such systems due to their stimuli‐responsive behaviour.[^4^, ^5^] At high pH, these materials self–assemble through non–covalent interactions to form micellar aggregates, such as worm–like micelles, spherical micelles, nanotubes, or fibrils.[^6^, ^7^] Due to the nature of their interactions, reversible changes in self–assembly or morphology can be obtained upon application of a variety of external triggers, such as light, electricity, pH or temperature.[^8^, ^9^, ^10^] Specifically, light is an attractive trigger to induce stimuli–responsive behaviour as it is non–invasive and can be applied with high spatiotemporal control, allowing the formation of patterned materials or surfaces. Photo–responsive systems comprising of LMWGs can be designed by appending photo–responsive moieties to the molecule or by introducing photo–responsive molecules in presence of the LMWG in solution.^[11]^ For this purpose, photoacids can be used to trigger gelation or aggregation responses by protonating pH‐responsive end groups under irradiation. For instance, metastable photoacids have been used to produce light–triggered supramolecular gels,[^12^, ^13^, ^14^] form supramolecular gels on photo–patterned surfaces,^[15]^ induce light–triggered reversible gel–to–sol transitions[^16^, ^17^, ^18^, ^19^] or generate reversible changes in supramolecular self–assembly or functionality for surfactant systems^[20]^ and DNA‐based systems.[^21^, ^22^] Here, we used a merocyanine photoacid **1** (Figure 1)^[23]^ to induce transient pH changes in a functionalised dipeptide solution and achieve reversible changes in viscosity. Under irradiation with visible light, merocyanine photoacids like **1** can isomerise to the ring–closed spiropyran form **2**, releasing a proton and leading to a significant pH drop.[^23^, ^24^, ^25^] In the case of photoacid **1**, this process can be repeated for at least 10 cycles, with minimal changes to the pH‐switching properties of the system.^[23]^ Based on these observations, we investigated the pH‐switching behaviour of photoacid **1** in the presence of functionalised dipeptide **1ThNapFF** (Figure 1).

**Figure 1:**
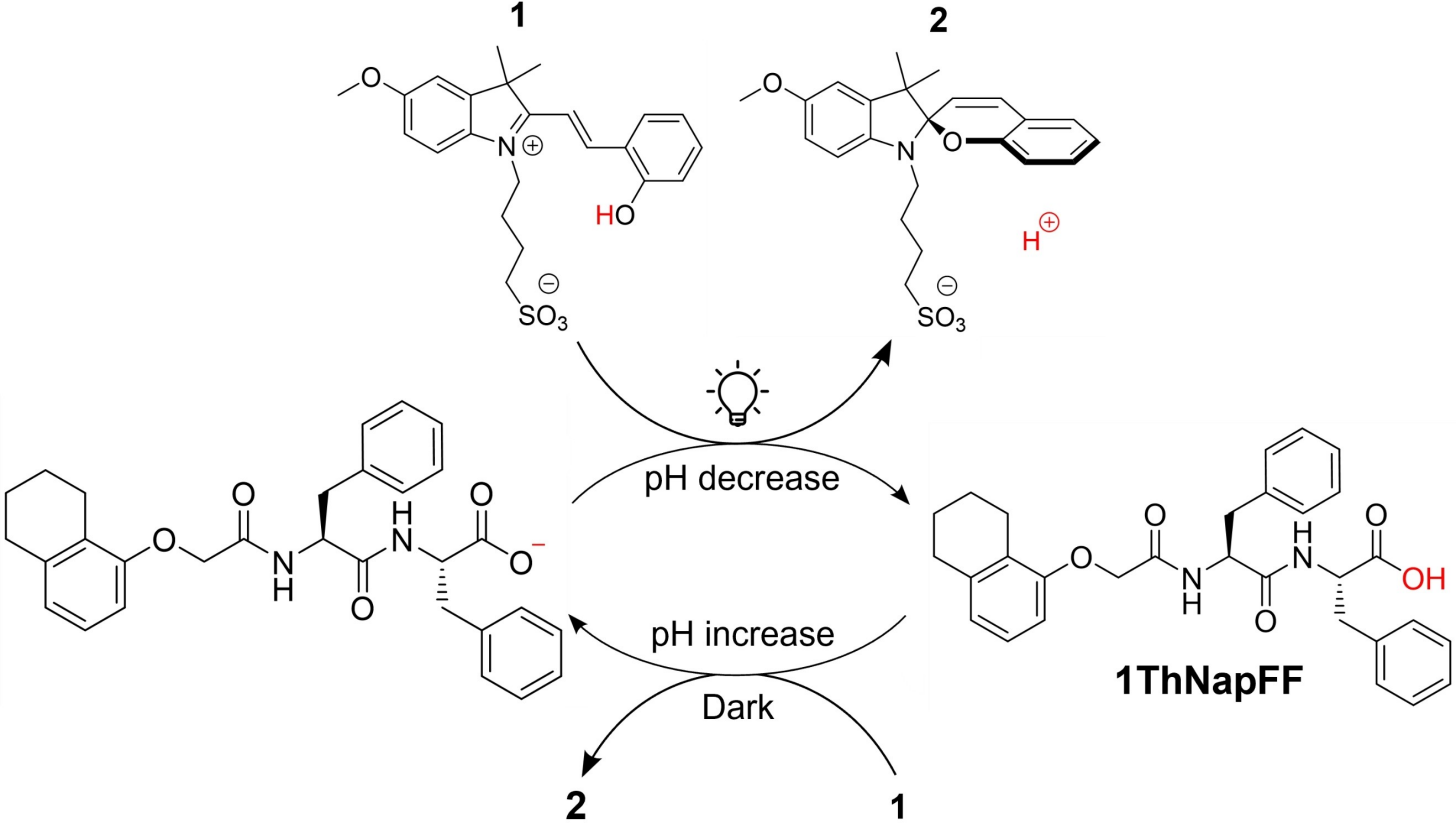
Schematic representation of the dynamic system presented in this work. Light–triggered isomerisation of merocyanine photoacid **1** to spiropyran **2** in presence of deprotonated **1ThNapFF** leads to a decrease in pH and protonation of the carboxylic acid moiety. In the dark, the system relaxes back to the initial pH, deprotonating **1ThNapFF** once more.

At high pH, **1ThNapFF** self–assembles into worm–like micellar structures.[^26^, ^27^] When the pH is decreased below the apparent p*K*_a_ of the gelator (~6.3),^[28]^ protonation of the carboxylic acid end leads to the formation of a network of fibres, often resulting in gelation.^[29]^ We show that photoacid **1** can be used to reversibly change the pH of the solution, leading to controllable dynamic changes in viscosity. A similar method of inducing reversible gel–to–sol transitions for a small dipeptide (**FF**) has been previously reported using a merocyanine photoacid.^[16]^ Compared to the previous system, here we show a system capable of switching viscosity in water–based solutions instead of organogels. Further, the presence of a solution phase throughout allows this system to be easily loaded in microfluidic cells, where the flow can be controlled on–demand using light. The pH switch here is induced within a larger window from a basic pH, allowing the system to be applicable to a wider range of materials. We have also shown characterisation of the system *in situ* across different length scales using a variety of techniques.

Solutions of **1ThNapFF** were prepared at 1.5 mg/mL (3 mM) at pH 7.2, followed by addition of photoacid **1** (1.5 mM) and KCl (20 mM, added to mimic preparation of photoacid solution as previously reported.^[23]^ The salt does not significantly affect the gelator behaviour (Figure S5, ESI)). The dipeptide concentration in this work was chosen due to the limited solubility of photoacid **1** (3 mM), ensuring dissolution of the photoacid up to 1 equivalent with respect to **1ThNapFF**. We note that this is lower compared to the concentration used for gelation of **1ThNapFF** (<2 mg/mL).^[26]^ Two concentrations of photoacid **1** were initially investigated to test the effect of the amount of protonation within the sample. With a photoacid **1** concentration equal to that of the gelator (3 mM), reversible viscosity increase was observed (Figure S6a, ESI). However, localised gelation domains did not allow homogeneous irradiation of the sample (Figure S6b, ESI), resulting in low control over the system light–triggered response as well as re–usability. Because of this, a lower concentration of 1.5 mM (0.5 eq. to the gelator) was used throughout the rest of this work. A decrease in viscosity was observed upon addition of photoacid **1** to the solution compared to **1ThNapFF** alone (Figure 2a). This suggests a potential interaction between the two components, leading to higher dissolution of the **1ThNapFF** molecule. This is further shown by small angle X‐ray scattering (SAXS, Figure 2b). At pH 7.2, an increase in the fibre radius from 10 nm to 12 nm is observed in the solutions containing the photoacid (Figure 2b, red data) compared to solutions without (Figure 2b, black data). In particular, the fits to the data show a decrease in lateral association of the fibres upon addition of **1**, indicating that the additive is potentially getting incorporated in the micellar structures and affecting the molecular packing of the dipeptide (Figures S9 and S10, Tables S1 and S2, ESI).

**Figure 2:**
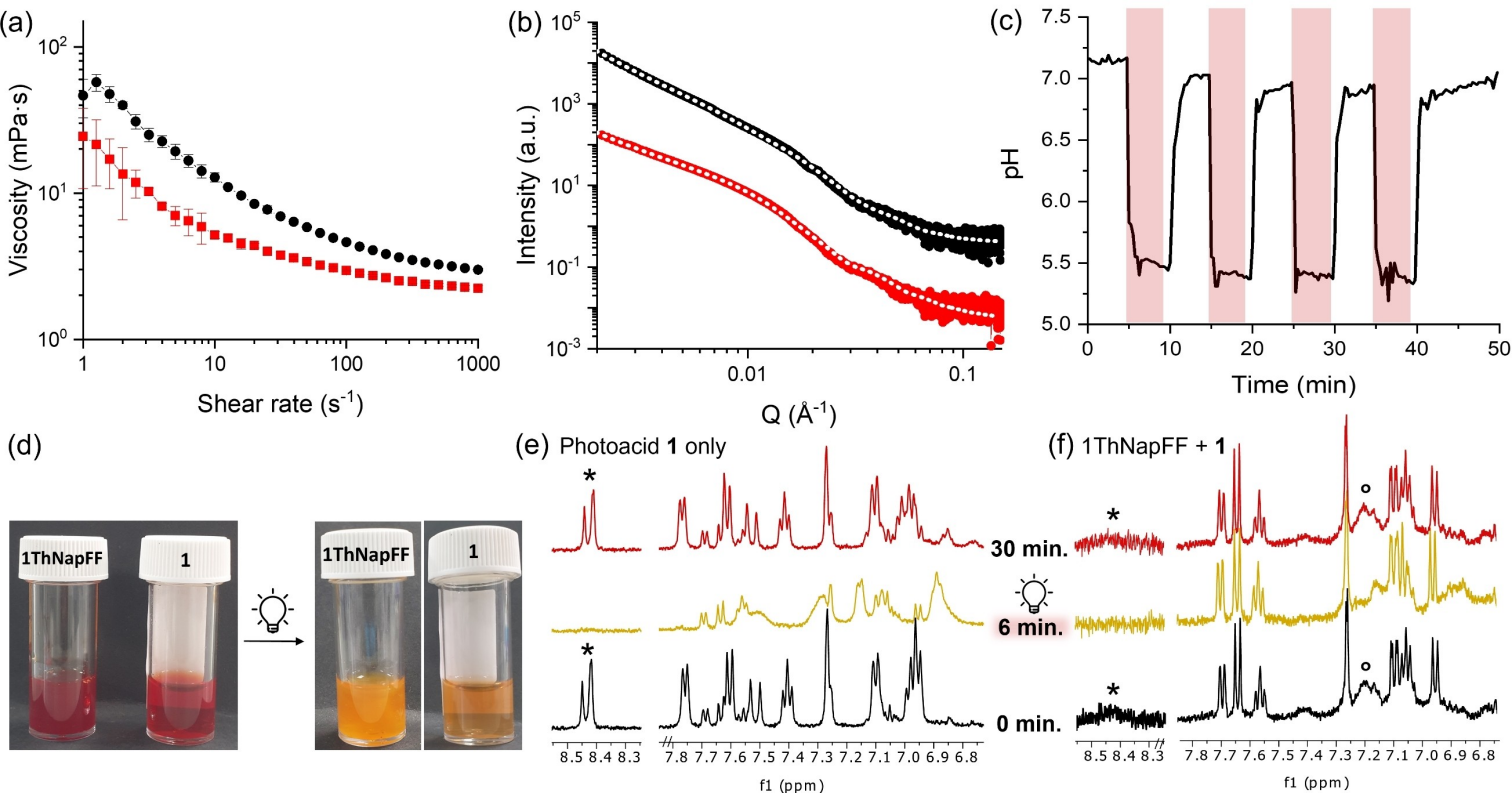
Viscosity (a) and SAXS data (b) of solutions of **1ThNapFF** (1.5 mg/mL) with photoacid **1** (1.5 mM) (red square) and without (black circle). Fits to the SAXS data (white dots) have been overlayed on the respective dataset; (c) pH‐switching behaviour for a solution of **1ThNapFF** (1.5 mg/mL) in presence of photoacid **1** (1.5 mM) over 4 irradiation cycles. The red–shaded areas indicate illumination with 450 nm LED; (d) photographs of solutions before and after irradiation of photoacid **1** (1.5 mM) with **1ThNapFF** (1.5 mg/mL) (left vial) and without (right vial); (e) NMR spectra of photoacid **1** in D_2_O (1.5 mM) prior to irradiation (black), after 6 minutes of irradiation (yellow) and after 30 minutes of relaxation in the dark (red), the peak corresponding to the characteristic alkene peak of **1** (*) is highlighted to show the change upon irradiation; (f) NMR spectra of a D_2_O solution of **1ThNapFF** (1.5 mg/mL) and photoacid **1** (1.5 mM) prior to irradiation (black), after 6 minutes of irradiation (yellow) and after 30 minutes of relaxation in the dark (red). The broad peaks corresponding to the characteristic alkene peak of **1** (*) and broad peak related to **1ThNapFF** (°) have been labelled. Full NMR spectra can be found in Figures S14–S18, ESI.

We then investigated the pH‐switching behaviour of the composite solution under irradiation with LED light at 450 nm. The pH drops from 7.2 to 5.3 upon turning on the light (Figure 2c), accompanied by a change in colour from red to yellow (Figure 2d). The **1ThNapFF** solution appears more turbid after irradiation (Figure 2d, left vial), suggesting further aggregation of the self‐assembled structure. The pH drop can be cycled for at least 4 consecutive cycles of 5‐minute irradiations (Figure 2c), with a similar pH reached in all repetitions (≈5.3). In the dark, the kinetics of pH recovery are slower than the light–induced pH drop, and the pH was found not to increase back to the initial value of 7.2 over the cycles. Longer relaxation times (30 minutes) are required for the pH to recover to values close to the initial pH, and a slightly lower pH is typically recorded (pH 7.1). This was also previously observed for light–responsive DNA assemblies with photoacid **1**.^[30]^ Potential interactions between the spiropyran **2** and the dipeptide could cause this slower relaxation. We note, however, that the pH is observed to fully recover to its initial value after overnight relaxation in the dark. Interestingly, no gelation was observed in the sample upon lowering the pH. This is likely because the kinetics of the ring–closing are faster than gelation kinetics or due to the low concentration of **1ThNapFF** used in this study. Nonetheless, a visual increase in viscosity was observed immediately after irradiation, indicating that the pH drop is leading to changes in aggregation.

To further understand this behaviour on the molecular and structural level, samples were irradiated *in situ* during NMR and SAXS were performed (Sections S1.5 and S1.7, ESI). In the dark, the NMR spectrum of the **1ThNapFF** and **1** solution shows several changes compared to the spectrum of only **1** (Figures 2e and 2 f, black spectra). The characteristic alkene doublet of photoacid **1** at 8.45 ppm broadens significantly in the presence of **1ThNapFF** (Figure 2f,*), suggesting interactions between **1** and **1ThNapFF**. Furthermore, a doublet centred at 6.95 ppm can be observed instead of a multiplet around 7.00–6.97 ppm. The doublet can be correlated to the signal of the alkene in spiropyran form **2**.^[23]^ It is therefore possible that **1** is significantly interacting with the dipeptide, resulting in more intense peaks from **2**, which might interact less with **1ThNapFF**. Upon irradiation, signals related to **1** disappear (Figures 2e, 2 f, yellow spectra, Figures S15, S17, ESI), agreeing with the literature data.^[23]^ Additionally, a broad peak assigned to **1ThNapFF** at 7.15 ppm in the composite solution is observed to disappear under irradiation (Figure 2f,°, Figure S17, ESI). The data suggest that fewer interactions occur between the photoacid molecule and the dipeptide when there is less **1** present in solution at low pH. As a result, **1ThNapFF** can aggregate further with itself, becoming NMR invisible. The NMR data show that this switch in assembly is reversible, as the peaks recover their intensity in the dark (Figures 2e, 2 f, red spectra, Figures S15, S17, ESI). Interestingly, SAXS data of the same solution showed no change in pattern under irradiation and in the dark (Figure 3a, yellow and red data, Section S2.2, ESI). The SAXS data here extends to around 300 nm, which is below the length of the fibres (<500 nm). It is likely that the interactions between the molecules are occurring at the network level and ultra–small angle X‐ray scattering is required to assess them.

**Figure 3:**
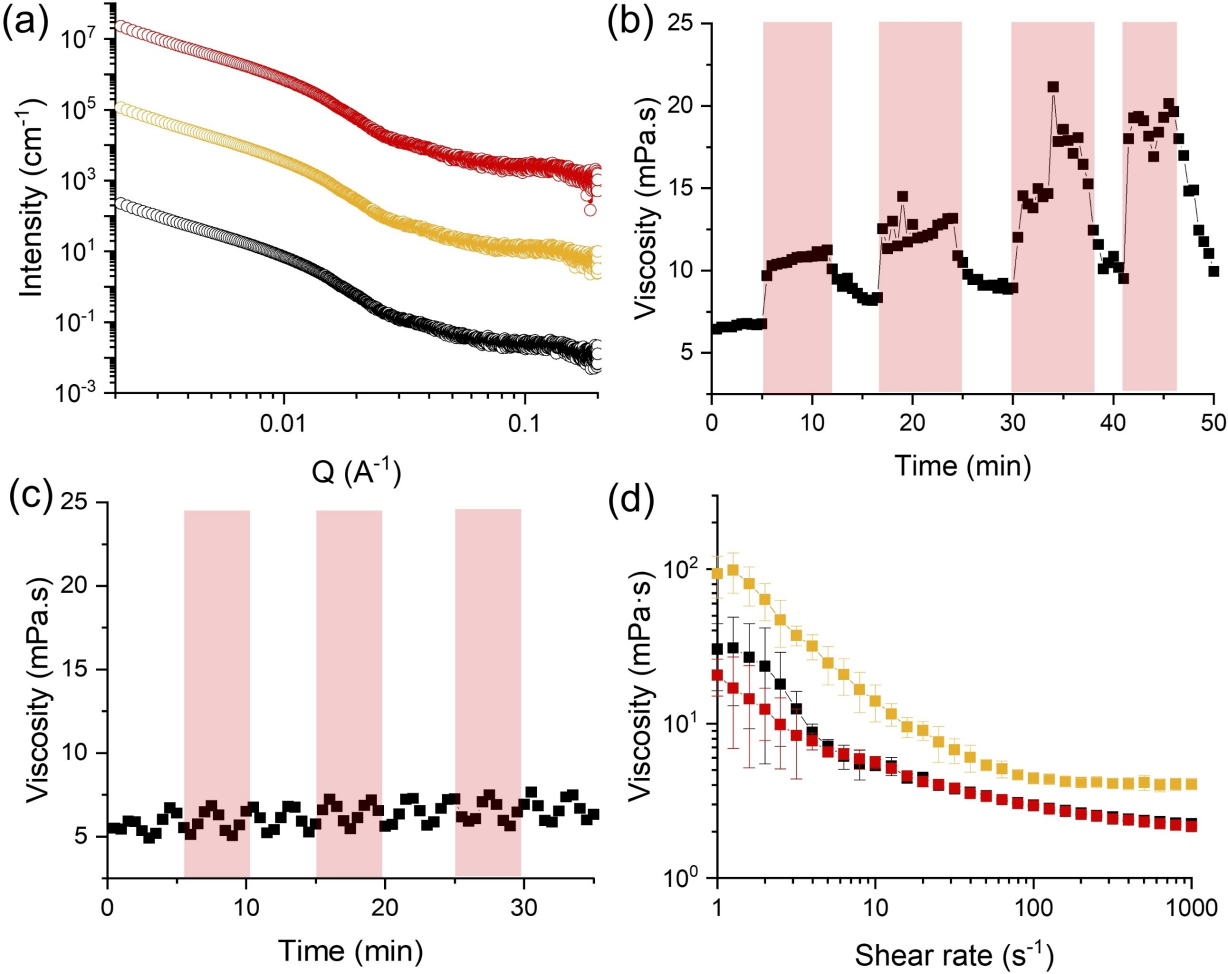
(a) SAXS data for a solution of **1ThNapFF** (1.5 mg/mL) with photoacid **1** (1.5 mM) in the dark (black), during irradiation (yellow), after relaxation (red). Constant shear viscosity under irradiation for a solution of **1ThNapFF** (1.5 mg/mL) (b) in presence of **1** (1.5 mM) and (c) without. (d) Viscosity data of solution of **1ThNapFF** (1.5 mg/mL) with photoacid **1** (1.5 mM) in the dark (black), during irradiation (yellow) and after relaxation (red). In all cases, red shaded areas indicate irradiation with 450 nm LED.

To observe the changes in viscosity over time, constant shear viscosity measurements were performed under irradiation using a rheo–irradiation set–up with a quartz glass bottom plate and a bespoke 3D‐printed LED holder (Section S1.3, ESI). The set–up was optimised to ensure that the sample measured was being irradiated with the light homogeneously. The sample was measured during 5 minutes of irradiation and then while recovering for 5 minutes in the dark. Under irradiation, the viscosity of the sample increases immediately (Figure 3b), staying at similar values until the LED is turned off. As the sample relaxes in the dark, the viscosity decreases back to values close to the initial value. The viscosity in the dark further increases over the course of the experiment. This can be ascribed to the fact that the de–aggregation of **1ThNapFF** might be slower than the pH recovery, resulting in higher protonation and aggregation over subsequent cycles. When no photoacid is added to a **1ThNapFF** solution, no changes in viscosity can be observed under irradiation (Figure 3c and Figure S8b, ESI). Similarly, no viscosity changes are seen in a photoacid–only solution upon irradiation (Figures S7c and S8a, ESI). To test the reproducibility of the viscosity increase, viscosity measurements on the **1ThNapFF** and **1** solution were run during irradiation (Figure 3d). In all cases, shear–thinning behaviour can be observed, consistent with the presence of worm–like micellar structures in all samples. The viscosity under irradiation increases (Figure 3d, yellow) and it relaxes back to its original value in the dark (Figure 3d, red). This confirms that the viscosity changes are due to the large light–induced pH drop.

Overall, the data indicate that we can reversibly induce increases in viscosity quickly and reversibly for 4 cycles on short timescales. By monitoring the temperature of the bottom plate of the rheometer, we can rule–out that such changes are induced by the increase in temperature upon irradiation (Section S2.1.3, ESI).

The observed behaviour can be used to control flow non–invasively by irradiation at specific locations. In this endeavour, a custom set–up was built to monitor changes in flow under irradiation (Figure 4a, Section S1.6, ESI). The set–up consists of a syringe pump fitted with transparent tubing, connected to a pressure gauge *via* a t‐piece. The pressure gauge is placed before the LEDs, allowing us to measure the pressure of the solution as it flows prior to irradiation. At rest with no sample flowing, the pressure gauge detects a pressure of 0.7 mbar (Figure 4b). When the sample is pumped through the system (1 mL/min) in the dark, an increase in the detected pressure is observed to a constant value of around 1.8 mbar. Upon irradiation, a rapid increase in pressure is detected to 4.4 mbar (Figure 4b, red shaded area), ascribed to the increase in viscosity of the solution. In the dark, the pressure slowly reverts to 1.9 mbar as the sample relaxes. Using the same solution, a similar increase in pressure under irradiation was observed for at least two repeats (Figure S19, ESI), indicating the reproducibility of this set–up. We further show that this behaviour can be used to stop flow through a small needle under irradiation (Supporting Video).

**Figure 4:**
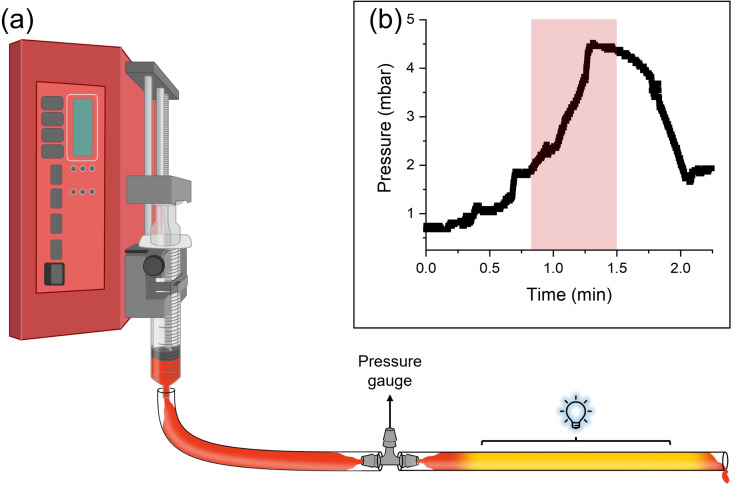
(a) Schematic representing the custom set–up to detect changes in pressure under irradiation used in this work. (b) Data collected for a solution of **1ThNapFF** (1.5 mg/mL) and **1** (1.5 mM) using the custom set–up. The red shaded area indicates irradiation with LED light (λ=450 nm).

In conclusion, we can combine the stimuli–responsive behaviour of a functionalised dipeptide with a photoacid capable of fast pH‐switching. This allowed us to design a system capable of reversible changes in viscosity. Our data show that interactions between the dipeptide and the photoacid are responsible for a decrease in viscosity, which in turn increases once the merocyanine photoacid isomerises to its ring–closed form. The change in pH as well as viscosity is reversible and reproducible for at least 4 irradiation cycles, with the sample relaxing to a lower viscosity in the dark. This method provides a facile way to design systems where flow can be controlled non–invasively and with a high level of spatial control. Such systems showing reversible viscosity changes are interesting for applications within microscale devices. For instance, these responsive systems can be used to direct flow within a microfluidic environment and, specifically, generate photoresponsive microvalves that can be controlled by localised light–irradiation of the solution.[^31^, ^32^] The fast kinetics of the system shown here would allow precise control of the opening and closing of the valves. The current challenge within these two‐component systems is related to the solubility of the photoacid molecule, as it can limit the concentration of self–assembling molecules that can be added to induce the changes. Improving the solubility of the photoacid would allow applications of such system to a wider variety of small self‐assembling molecules with different limits of dissolutions to achieve larger changes in viscosity. As a final point, the self–assembled structures formed by **1ThNapFF** persist at higher temperatures and heating and cooling can be used to modify the viscosity of these systems,^[33]^ meaning that there is the potential for using these self–assembled systems at higher temperatures.

## Supporting Information

The authors have cited additional references within the Supporting Information.[^34^, ^35^, ^36^, ^37^, ^38^, ^39^]

## Conflict of interests

The authors declare no conflict of interest.

## Supporting information

As a service to our authors and readers, this journal provides supporting information supplied by the authors. Such materials are peer reviewed and may be re‐organized for online delivery, but are not copy‐edited or typeset. Technical support issues arising from supporting information (other than missing files) should be addressed to the authors.

Supporting Information

Supporting Information

## Data Availability

The data that support the findings of this study are available in the supplementary material of this article.
