# Supplementary material for: Reversibly Tuning the Viscosity of Peptide‐Based Solutions Using Visible Light
Source: Chemistry. 2024 Mar 15;30(25):e202400544. doi: 10.1002/chem.202400544 (PMC11497239; doi:10.1002/chem.202400544)
Supplement: Supplementary file 1 — Supporting Information [file CHEM-30-e202400544-s001.pdf]

Chemistry–A European Journal

Supporting Information

Reversibly Tuning the Viscosity of Peptide-Based Solutions Using Visible Light

Simona Bianco, Laura Wimberger, Yael Ben-Tal, George T. Williams, Andrew J. Smith, Jonathon E. Beves, and Dave J. Adams*

Reversibly tuning the viscosity of peptide-based solutions using visible light

Simona Bianco,[a] Laura Wimberger,[b] Yael Ben-Tal,[c] George T. Williams,[c,d] Andrew J. Smith,[e] Jonathon E. Beves,[b] Dave J. Adams*[a]

Supporting information

1 Materials and methods

1.1 Materials

1ThNapFF was synthesised as previously been reported.[34] The merocyanine photoacid **1** was synthesised as previously reported.[23] All other reagents and solvents were purchased from Sigma-Aldrich and Alfa Aesar and used as received without further purification. De-ionised water was used throughout.

1.2 Solution preparation

1.2.1 1ThNapFF solutions

To prepare aqueous solutions of 1ThNapFF without photoacid, 1ThNapFF was weighed into a 7 mL Sterilin vial, followed by deionised water and 1 equivalent of 0.1M KOH to achieve a final concentration of 1.5 mg/mL. Typically, 5 mL of solution were prepared for ease of handling. The solutions were stirred overnight at 1000 rpm. The pH of the solution was adjusted up to 11.0 ± 0.1 using 1M KOH to ensure dissolution of any micellar aggregates. Then, the pH was brought down to 7.2 ± 0.1 using 1M HCl. During pH adjusting, the solutions were stirred for at least 30 minutes after addition of acid to ensure homogeneity as insufficient mixing could result in localised pH differences. If localised gelation occurred, samples were left to stir for at least 1 hour until full dissolution could be observed. KCl was added to the solution under stirring to achieve a final concentration of 20 mM. Solutions were prepared fresh every day for each measurement and the pH was checked before use to ensure a starting pH of 7.2 ± 0.1.

1.2.2 1ThNapFF and photoacid solutions

1ThNapFF solutions in presence of photoacid were prepared in a similar way as described in 1.2.1. The solution was then added to a pre-weighed amount of photoacid **1** to achieve a final concentration of 1.5 mM. The solution was stirred at 1000 rpm for 30 minutes or until full dissolution of **1**. The pH was then checked to ensure an initial pH of 7.2 ± 0.1 and adjusted if needed. The pH did not change significantly after addition of **1**. For solutions of **1** without 1ThNapFF, **1** was weighed in 7 mL Sterilin vials, followed by addition of 20 mM aqueous KCl solution (H_2O) to a final concentration of 1.5 mM. The solution was stirred for 30 minutes or until full dissolution at 1000 rpm. The pH was checked and adjusted to an initial pH of 7.2 ± 0.1 using either 0.1M KOH or 0.1M HCl. In all cases, to avoid interaction with light, the solutions were wrapped in tinfoil.

1.3 Viscosity measurements

Viscosity measurements of the samples prior to irradiation and during irradiation were performed using an Anton Paar Physica MCR302 rheometer with a temperature-controlled quartz bottom plate. To irradiate the sample, a 450 nm LED (0.7 A, RS Components Ltd) was held in place under the quartz bottom plate using a bespoke 3D-printed holder (Figure S1b). The set-up is shown in Figure S1a and S1b below. The intensity of the light was measured using a Thorlabs Optical Power Meter PM100D and Thorlabs sensor S/N: 16100711 to test the intensity of the light across the quartz plate. Based on the results, the intensity of the light was homogeneous at around 21 mW across a diameter of 30 mm. Hence, a CP25 geometry (cone angle 1°) was

used to measure the viscosity of the samples in this study. As an example, a picture of the sample after irradiation is shown in Figure S1c, which looks yellow throughout, indicating homogeneous irradiation.

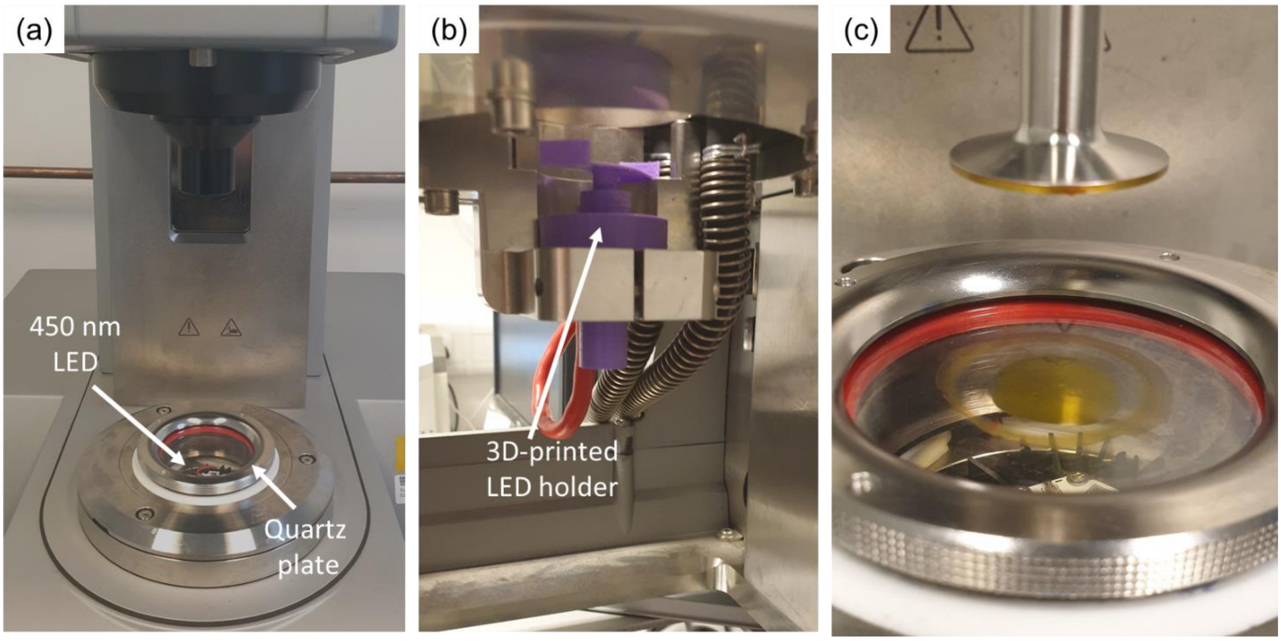

Figure S1. (a), (b) Irradiation set-up on the Anton Paar Physica MCR302 rheometer with a 450 nm LED powered by a constant-current power source. The pictures show the bespoke 3D-printed LED holder placed under the quartz plate. (c) Picture of a solution of 1ThNapFF (1.5 mg/mL) and **1** (1.5 mM) after 30 seconds of irradiation under the CP25 geometry showing a homogeneous colour throughout.

For all viscosity measurements, all solutions were poured on the quartz bottom plate to avoid any shear-thinning. The top plate was lowered on the solution at a gap height of 0.047 mm and any excess was gently dabbed away with a small amount of paper towel. Dynamic viscosity was measured by collecting viscosity data at shear rates from 1 s^{-1} to 1000 s^{-1}. For the constant viscosity measurements under irradiation, data points were collected every 30 seconds at a constant shear rate of 10 s^{-1}. The LED was manually turned on after 5 minutes in the dark and then turned off after 5 minutes of irradiation. Care was taken in observing the temperature of the rheometer bottom plate to observe any sharp increases under irradiation. This is shown in Section 2.1.1.

1.4 pH measurements

pH measurements under irradiation were performed using a HANNA FC200 pH probe with a 6 mm x 10 mm conical tip with an accuracy of ± 0.1. The pH change of the system was monitored under irradiation using a custom-made set-up in a dark box, shown below (Figure S2). The sample was positioned on a bespoke 3D-printed holder, with two LEDs (450 nm, 0.7 A, RS Components Ltd) placed at 1 cm away from the sample.

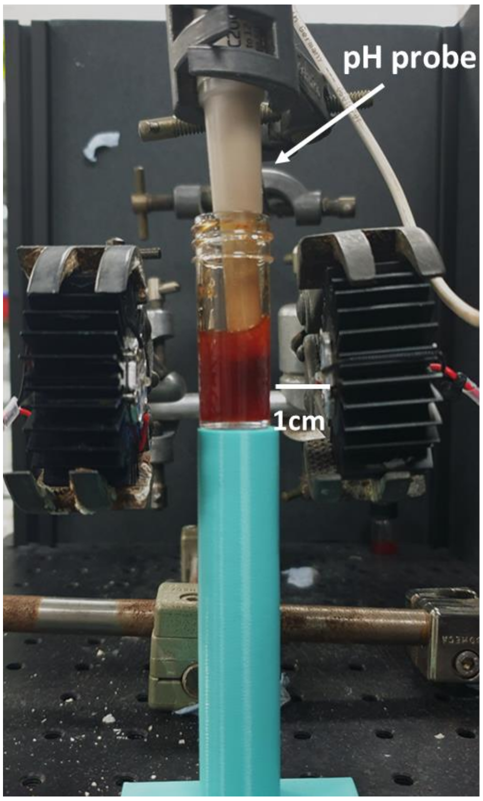

Figure S2. Custom set-up to measure pH changes under irradiation in a dark box. The sample is placed on a bespoke 3D-printed holder. The LEDs are placed at 1 cm away from the sample and a pH probe is inserted in the vial.

1.5 ^{1}H NMR Measurements

In situ ^{1}H NMR irradiation experiments were performed using a Bruker AVIIIHD500 FT-NMR spectrometer equipped with a Bruker BBFO CryoProbe at a constant temperature of 298 K, using an irradiation set-up based on a previous design by Gshwind and co-workers and closely resembling one previously reported by Ben-Tal and Lloyd-Jones.[35,36] A Thorlabs mounted LED (470 nm maxima, M470L5), powered by Thorlabs T-cube LED driver was coupled to a length of FP1500URT cable, the other end of which was stripped to a length of ≈ 3 cm and roughened with sandpaper to ensure even light distribution. The stripped end was placed inside a quartz coaxial insert (Norrel scientific), which was then inserted into 280 μL of the experimental solution inside an amberized 5 mm NMR tube; this corresponds to a light pathlength of 0.44 mm.[36] The T-cube power source was set to the maximum current for the M470L5 LED. The LED source and NMR console were connected via a split BNC cable to a Prizmatic PulserPlus TTL signal generator, which was activated via an external trigger switch, to ensure that spectra collection and illumination began simultaneously.

1.6 Syringe pump set-up

A ProSense single channel syringe pump was used to monitor the flow of the 1ThNapFF and **1** solution. A 12 mL syringe was used, attached *via* Luer lock fittings to a PVC tubing with a 3mm inner diameter. The tubing was cut in the middle and connected to a custom-built manometer *via* a t-piece and rubber tubing. The high precision manometer "Cavitation Rheometer Analyser Box (CRAB)"[37] has data logging capability to record

the pressure in the system. The syringe was loaded directly with the gelator solution by removing the plunger and pouring the sample in to avoid any shear-thinning of the solution. The syringe was then placed in the syringe pump and attached to the tubing. Note here that, although not pictured, the tubing was held by a bespoke 3D-printed holder to ensure that the tubing was at the same height throughout. At the end of the tubing, a beaker was placed to collect the solution. To keep the sample in the dark, a large box was used to cover the whole set-up. The collection of pressure data points was started prior to any flow. Next, the solution was allowed to flow through the system at a rate of 1 mL/min. Once the solution travelled through the whole system, the LEDs were turned on to collect the data during irradiation.

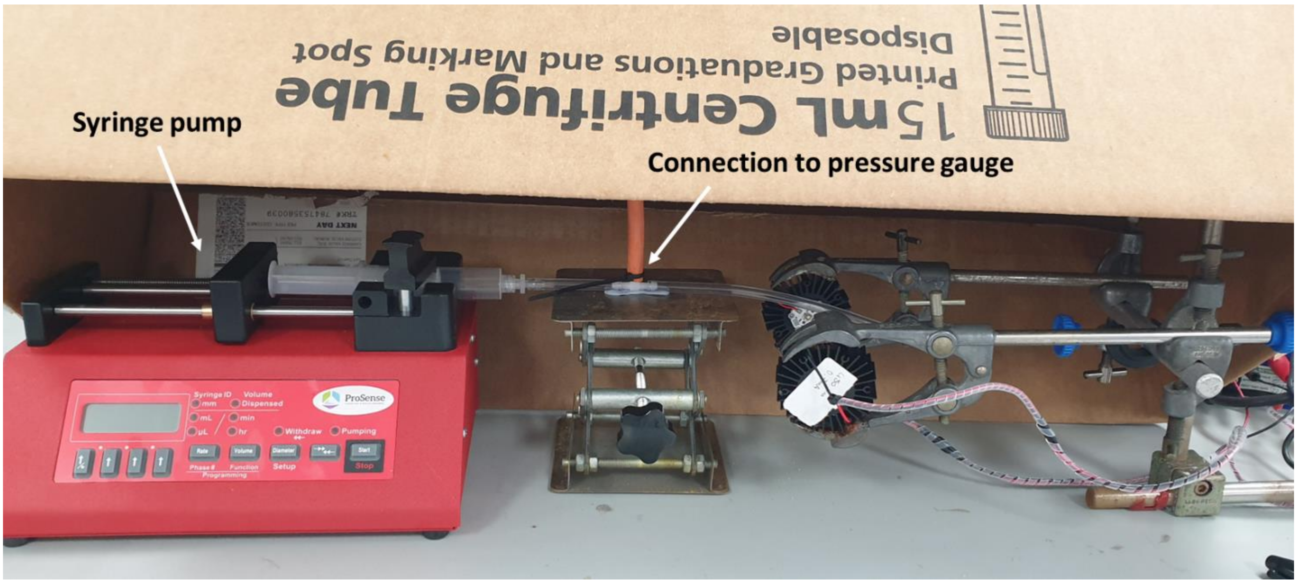

Figure S3. Custom set-up to measure changes in pressure under irradiation with 450 nm LEDs as the sample flows through a tubing system.

1.7 Small angle X-ray Scattering

Small angle X-ray scattering experiments were conducted at the I22 beamline at Diamond Light Source (Didcot, UK) with experiment number SM33006-1. The beamline operates at an energy of 12.4 keV and the camera length was set at 8.756 m to achieve a q range of 0.00174 – 0.2036 Å^{-1}. The solutions were prepared as previously described and loaded in quartz capillaries using a 1 mL syringe and 21G needle. The capillaries were then capped and wrapped in tinfoil prior to beamtime. To measure the samples under irradiation, an *in situ* irradiation sample environment was designed (Figures S4a and S4b). The set-up consisted in a LED holder positioned at a fixed height (Figure S4a), which was placed under a capillary holder (Figure S4c). The distance between the LED and the capillary could be adjusted and it was fixed at 1 cm. This allowed the sample to be irradiated homogeneously, with a change in colour observed throughout the capillary for at least 30 seconds of irradiation. However, we note that longer irradiations (5 minutes) combined with the X-ray data collection appeared to cause bleaching of the photoacid: the sample was not able to relax back to the initial state in the position of the data collection (Figure S4c, yellow area) even after leaving the sample for 16 hours in the dark.

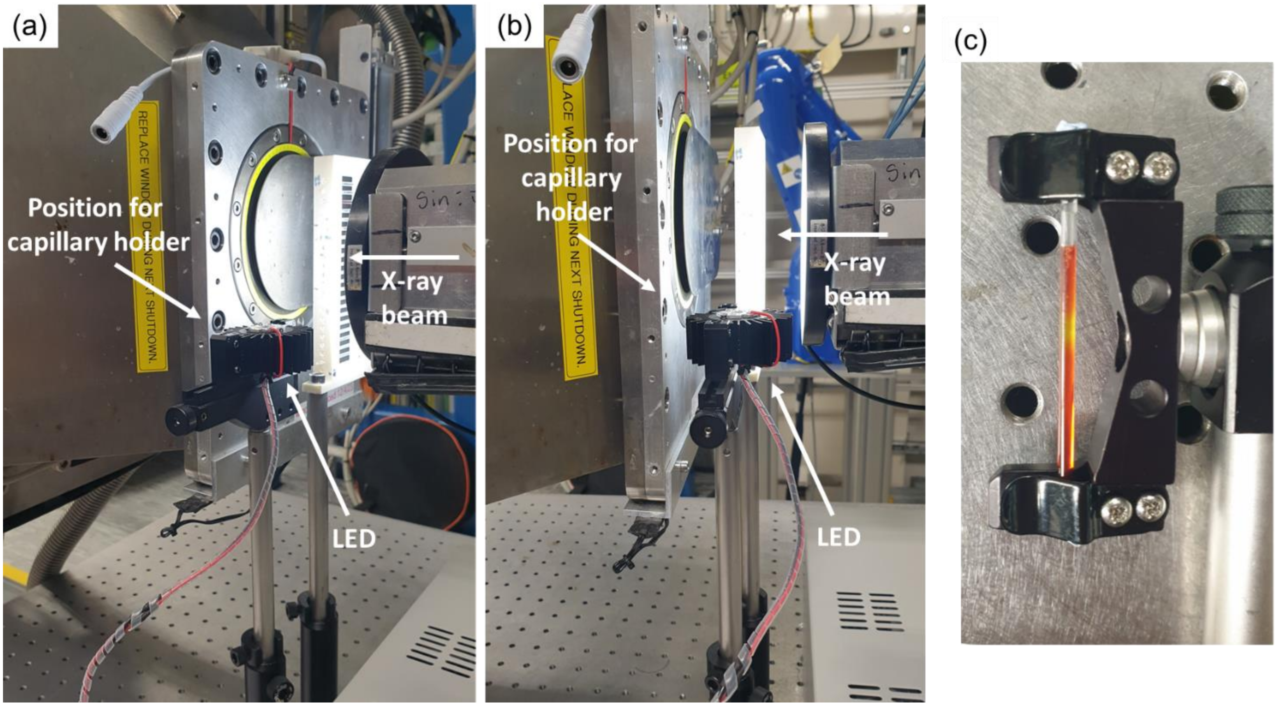

Figure S4. (a), (b) Photographs of the sample environment used at Diamond Light Source to test morphology changes under irradiation. The LED is held on a mount at a fixed height with a capillary holder placed above it. The distance between capillary and LED could be fixed by changing the fixed height of the post collar. (c) Picture of the capillary holder used in this set-up. The capillary contains a solution of 1ThNapFF and **1** after repeated irradiation cycles (5 minutes). The sample shows inhomogeneity in colour due to bleaching of the photoacid in the area where data collection was performed. After 16 hours, the sample was not able to relax back to the initial colour. This did not occur for irradiation periods of 30 seconds.

For all samples, 100 x 10 ms frames were collected and averaged. All samples were kept in the dark for 5 minutes, irradiated for 30 seconds and left to relax for 5 minutes. This was repeated for 4 cycles for all solutions. Note here that measurements were also carried out for a sample of 1ThNapFF and **1** with a longer irradiation time of 5 minutes. However, no significant changes were observed for this sample (Figure S12, Section 2.2.1), with an area of the sample not being able to relax back within the capillary even after prolonged times of 16 hours (Figure S4, right). To avoid this, shorter irradiation times were used for the samples, as the viscosity changes can be already observed within 30 seconds of irradiation.

The data was processed using Dawn Science (version 2.27),[38] according to a standard I22 pipeline.[39] As part of the processing, the solvent background (H_2O) was subtracted and a full azimuthal integration was performed to yield I vs q plots, which were fitted to models using SasView (version 5.0.2). For this, scattering length densities (SLDs) were calculated using the NIST neutron activation and scattering calculator (https://www.ncnr.nist.gov/resources/activation/), assuming a density of 1.58 g/cm^3 for the dipeptide. For 1ThNapFF, a solvent SLD of 14.025x10^{-6} Å^2 was used, while for H_2O a solvent SLD of 9.469x10^{-6} Å^2 was used.

2 Results

2.1 Rheology

2.1.1 Viscosity measurements with 0 mM KCl

To test the effect of the presence of salt in the solution on the behaviour of the gelator, shear viscosity measurements were run on a sample containing 1ThNapFF (1.5 mg/mL) and no KCl (Figure S5a, black data). The data shows no significant changes compared to a sample containing 20 mM KCl (Figure S5a, red data). The reversible viscosity change was further tested in a solution of 1ThNapFF (1.5 mg/mL) and **1** (1.5 mM) with 0 mM KCl (Figure S5b) and a viscosifying behaviour was still observed.

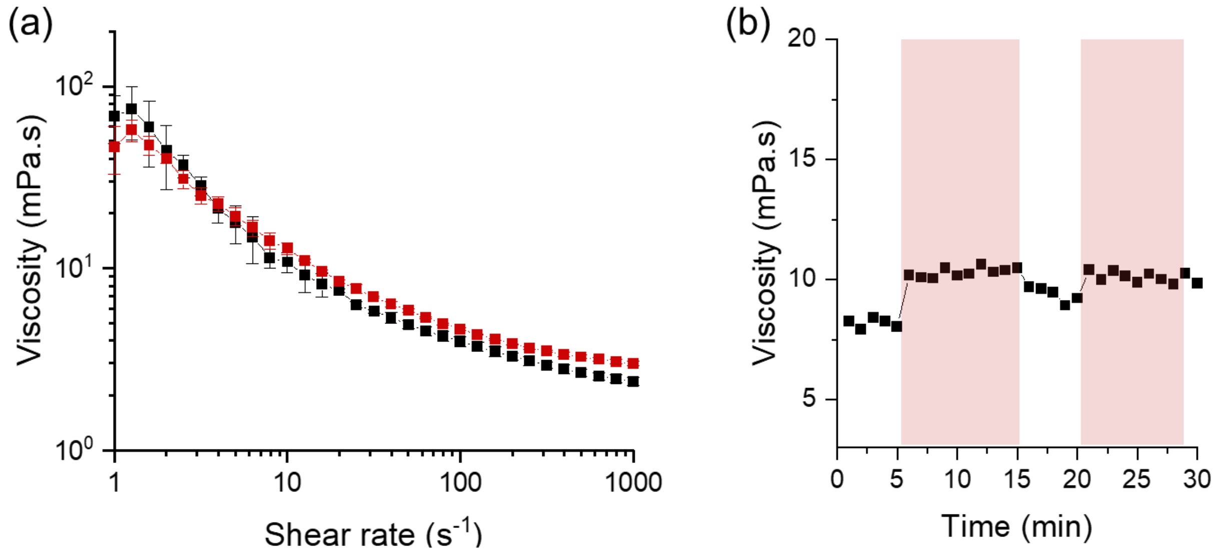

Figure S5. (a) Shear viscosity data for a solution of 1ThNapFF (1.5 mg/mL) at pH 7.2 in presence of 20 mM KCl (red data) and 0 mM KCl (black data). (b) Constant shear viscosity measurement under irradiation of a solution of 1ThNapFF (1.5 mg/mL) and **1** (1.5 mM) with 0 mM KCl. The red-shaded areas indicate irradiation with 450 nm light.

2.1.2 Constant viscosity measurements with temperature

To test the effect on photoacid concentration in the sample, a solution containing 1ThNapFF and **1** was prepared at 1 eq. of photoacid (3 mM). As for the 1.5 mM, a reproducible increase in viscosity can be observed upon irradiation, with higher viscosities compared to the previous solution (Figure S6a). However, the data progressively appears to be noisier over each cycle. This is potentially due to localised gelation occurring within the sample (Figure S6b). The presence of these opaque domains also did not allow the sample to be homogeneously irradiated over subsequent cycles. Because of this behaviour and the limited control over this localised gelation, the photoacid concentration was limited to 1.5 mM throughout the rest of this work.

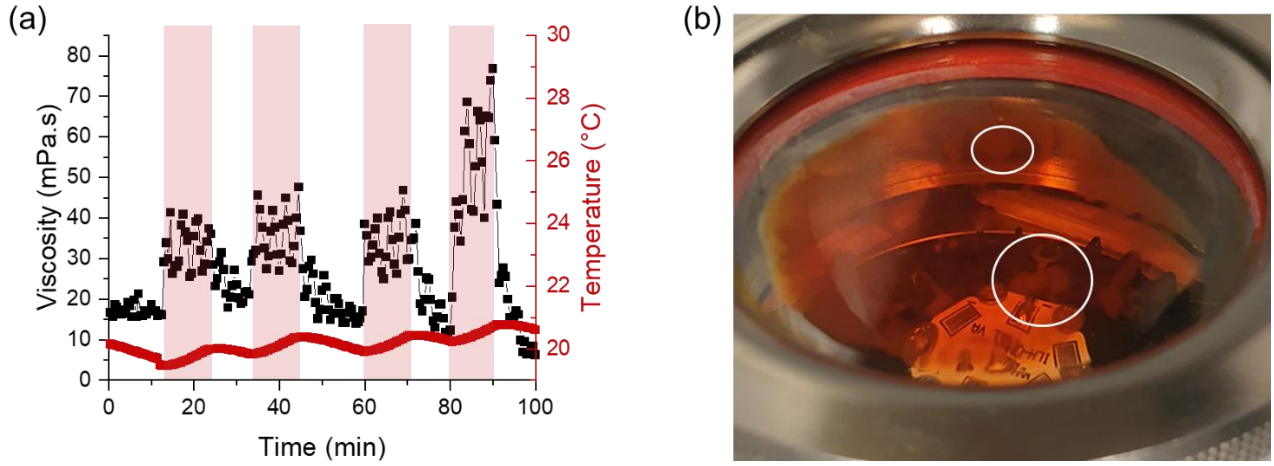

Figure S6. (a) Constant shear viscosity measurement recorded under irradiation for a solution 1ThNapFF (1.5 mg/mL) and **1** (3 mM). The red shaded areas indicate irradiation of the sample with 450 nm light. (b) Image of a solution of 1ThNapFF (1.5 mg/mL) and **1** (3 mM) after the irradiation cycles, highlighting the presence of localised gel domains within the solution (white circles).

2.1.3 Constant viscosity measurements with temperature

The influence of temperature on the viscosity increase on a sample was monitored by collecting the temperature values of the bottom plate of the rheometer. In all cases, a very small increase in temperature (~1°C) over time can be observed over repeated irradiation (Figure S7). No increases in viscosity can be observed for samples with just the gelator (Figure S7b) and just the photoacid (Figure S7c) showing that the temperature does not affect sample viscosity and the observed changes are due to the fast pH dropping of the photoacid in presence of gelator.

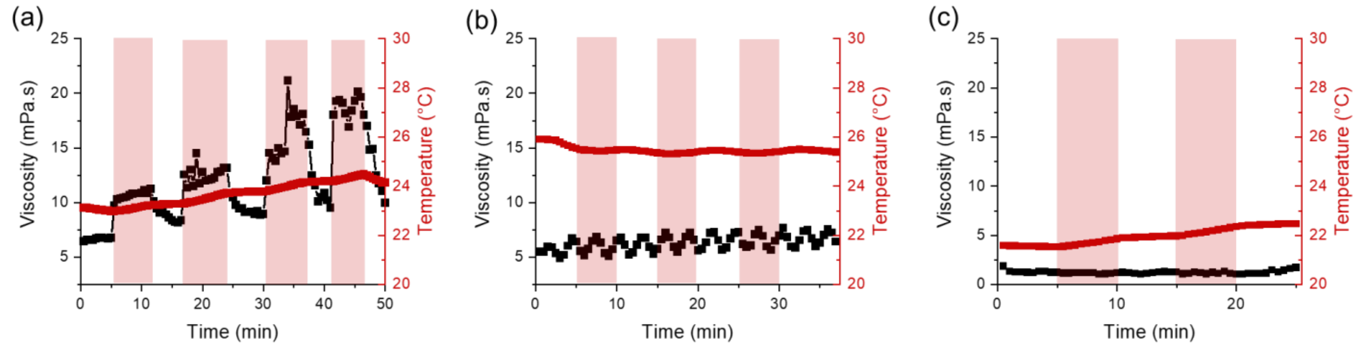

Figure S7. Constant shear viscosity measurements recorded under irradiation for (a) a solution of 1ThNapFF and **1**, (b) 1ThNapFF only and (c) photoacid **1** only. In all cases, final concentrations of 1ThNapFF and **1** are 1.5 mg/mL and 1.5 mM. The red shaded areas indicate irradiation of the sample with 450 nm light, the black squares indicate the viscosity and red squares indicate temperature.

2.1.4 Shear viscosity

The shear viscosity of solutions of just 1.5 mg/mL 1ThNapFF and of just 1.5 mM **1** were investigated after 5 minutes of irradiation. It can be observed that there is no significant change in viscosities of the samples after irradiation (Figure S8, yellow squares) and the data is reproducible across samples.

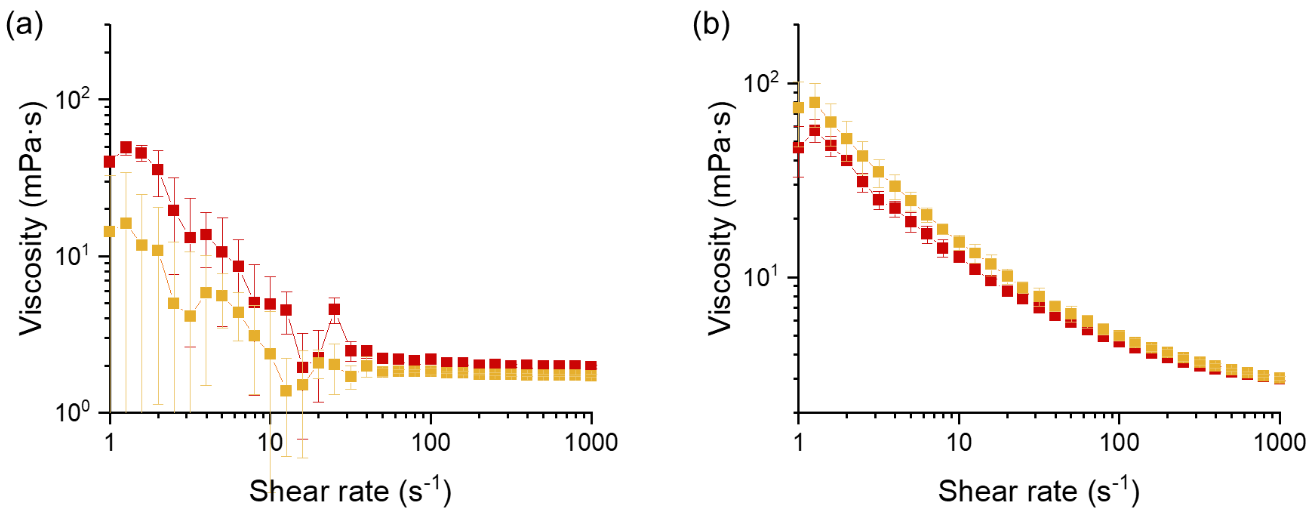

Figure S8. Viscosity measurements of a solution containing (a) only photoacid **1** (1.5 mM) and (b) only 1ThNapFF (1.5 mg/mL). The red squares indicate the data prior to irradiation and the yellow squares indicate data after irradiation. The error bars are calculated from triplicate experiments.

2.2 Small angle X-ray Scattering

All samples tested showed no significant change in SAXS patterns upon irradiation. The samples containing 1ThNapFF both could be fit to elliptical cylinder models combined with a power law model, consistent with the presence of worm-like micellar structures in the system. The elliptical cylinder model could be ascribed to some lateral association of fibres or bundles, as at pH 7.2 we might expect interactions between the micelles due to the closeness of the pH value to the pK_a. For the solution containing 1ThNapFF and **1**, the data could be fit to an elliptical cylinder model with a radius of 12.0 nm and an axis ratio of around 2 (Figure S9, Table S1). The data for the solution containing just 1ThNapFF was more difficult to fit, as Sasview would converge to models that were not able to fully capture the bump in the data at around 0.03 Å^{-1}. Hence, values were manually added to the fit to obtain data that best followed the scattering curve, which resulted in an elliptical cylinder model with a radius of 10.0 nm. An axis ratio between 3 and 4 provided a good fit to the data, with the value of 3.5 capturing the data well (Figure S10, Table S2). This can be related to some polydispersity in the system and the size of the aggregates formed in solution. The difference between the fits of the two samples could suggest that **1** interacts with 1ThNapFF such that less association between dipeptide micelles occurs. No significant scattering was instead observed for the sample containing just photoacid **1** and the data could be simply fit to a power law model (Figure S11, Table S3).

All datasets were fit to the same fit curve to highlight the similarities in scattering data. Minor decreases in intensity of the data can be observed over time, which could be ascribed to X-ray damaging over the course of the measurements.

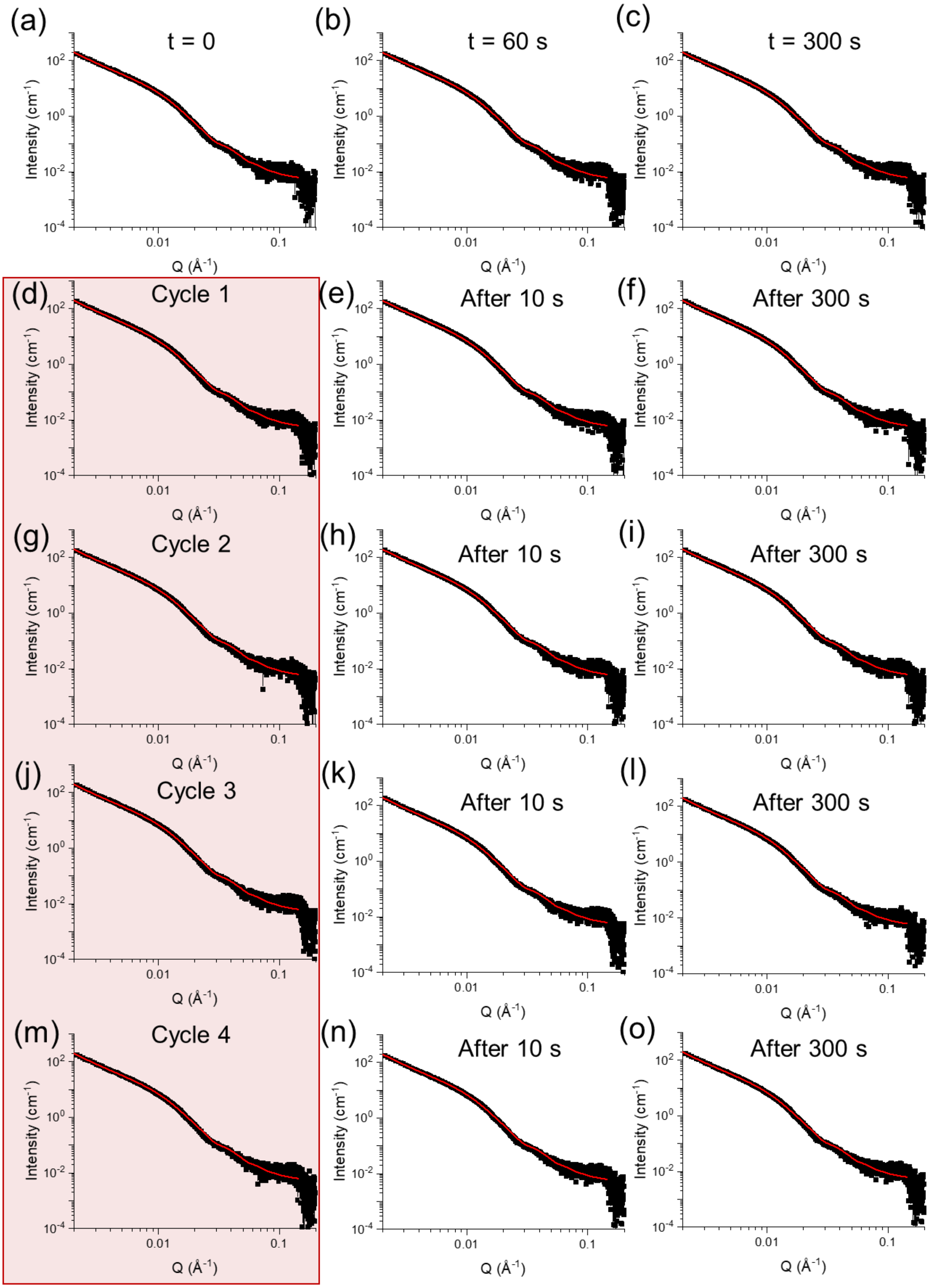

Figure S9. SAXS data (black squares) with model fits (red lines) for a solution 1ThNapFF (1.5 mg/mL) and photoacid **1** (1.5 mM) in the dark at pH 7.2 (a) – (c), after 30 seconds of irradiation (d), (g), (j), (m) and after 5 minutes of relaxation (e) - (f), (h) – (i), (n) – (o) over 4 cycles. The red square indicates samples that were measured under irradiation.

Table S1. Fitting parameters for SAXS data in Figure S9. The data was fit to an elliptical cylinder (A) combined with a power law model (B). Values that were manually added and fixed have been labelled with *.

1ThNapFF + **1**	**Value**	**Error**
**Background** / cm^{-1}	0.0063*	/
**A_scale**	$2.18x10^{-4}$	$1.74x10^{-6}$
**A_radius_minor** / Å	119.73	0.63
**A_axis_ratio**	2.08	0.018
**A_length** / Å	5000*	/
**B_scale**	$5.77x10^{-6}$	$3.19x10^{-7}$
**B_power**	2.73	0.009
χ^2	1.12	

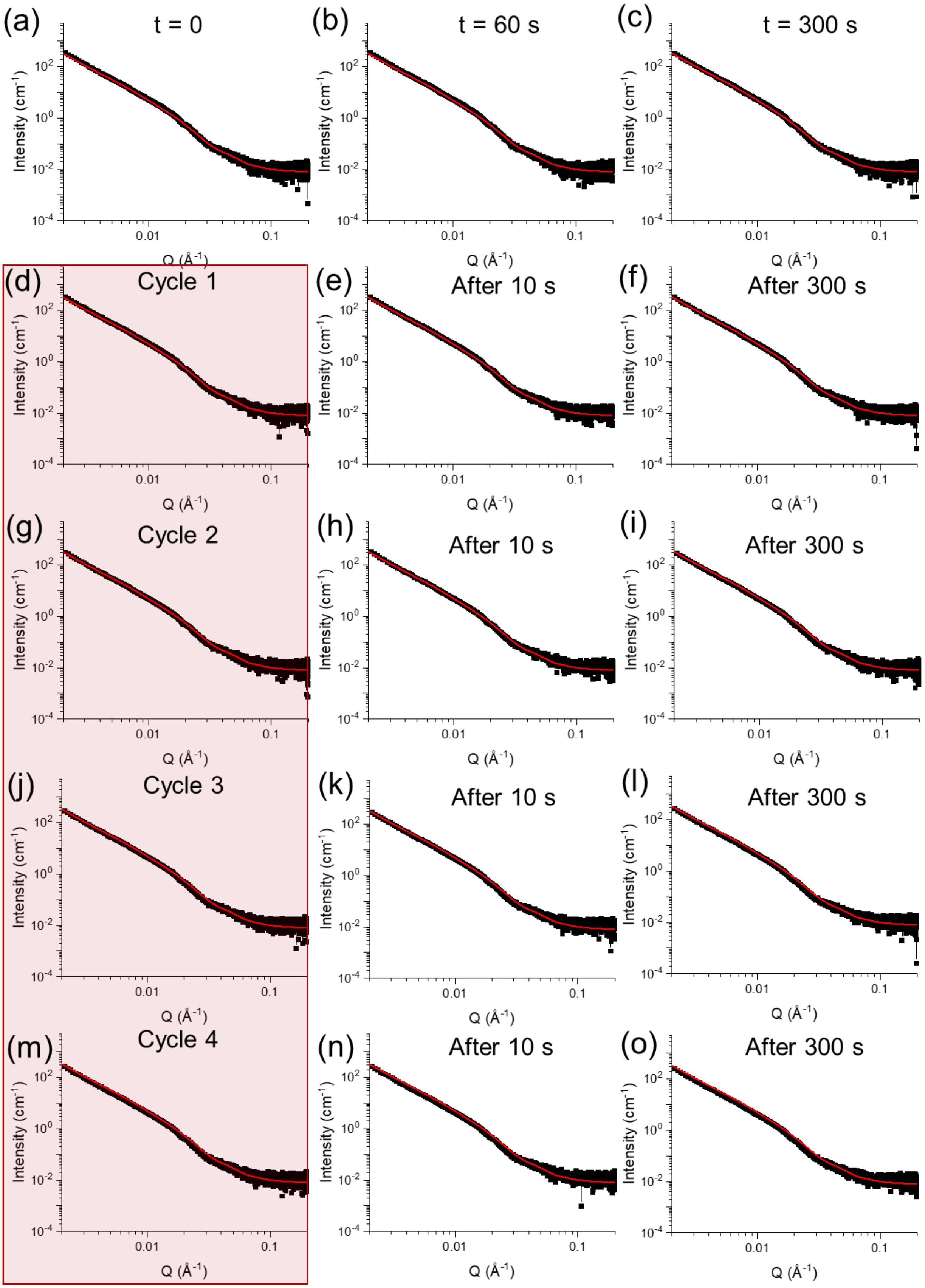

Figure S10. SAXS data (black squares) with model fits (red lines) for a solution 1ThNapFF (1.5 mg/mL) in the dark at pH 7.2 (a) – (c), after 30 seconds of irradiation (d), (g), (j), (m) and after 5 minutes of relaxation (e) - (f), (h) – (i), (n) – (o) over 4 cycles. The red square indicates samples that were measured under irradiation.

Table S2. Fitting parameters for SAXS data in Figure S10. The data was fit to an elliptical cylinder (A) combined with a power law model (B). Values that were manually added and fixed have been labelled with *.

1ThNapFF	**Value**	**Error**
**Background** / cm^{-1}	0.008*	/
**A_scale**	$1.40x10^{-4}$	$3.30x10^{-7}$
**A_radius_minor** / Å	100*	/
**A_axis_ratio**	3.5*	/
**A_length** / Å	5000*	/
**B_scale**	$1.49x10^{-6}$	$1.90x10^{-9}$
**B_power**	3.1*	/
χ^2	1.73	

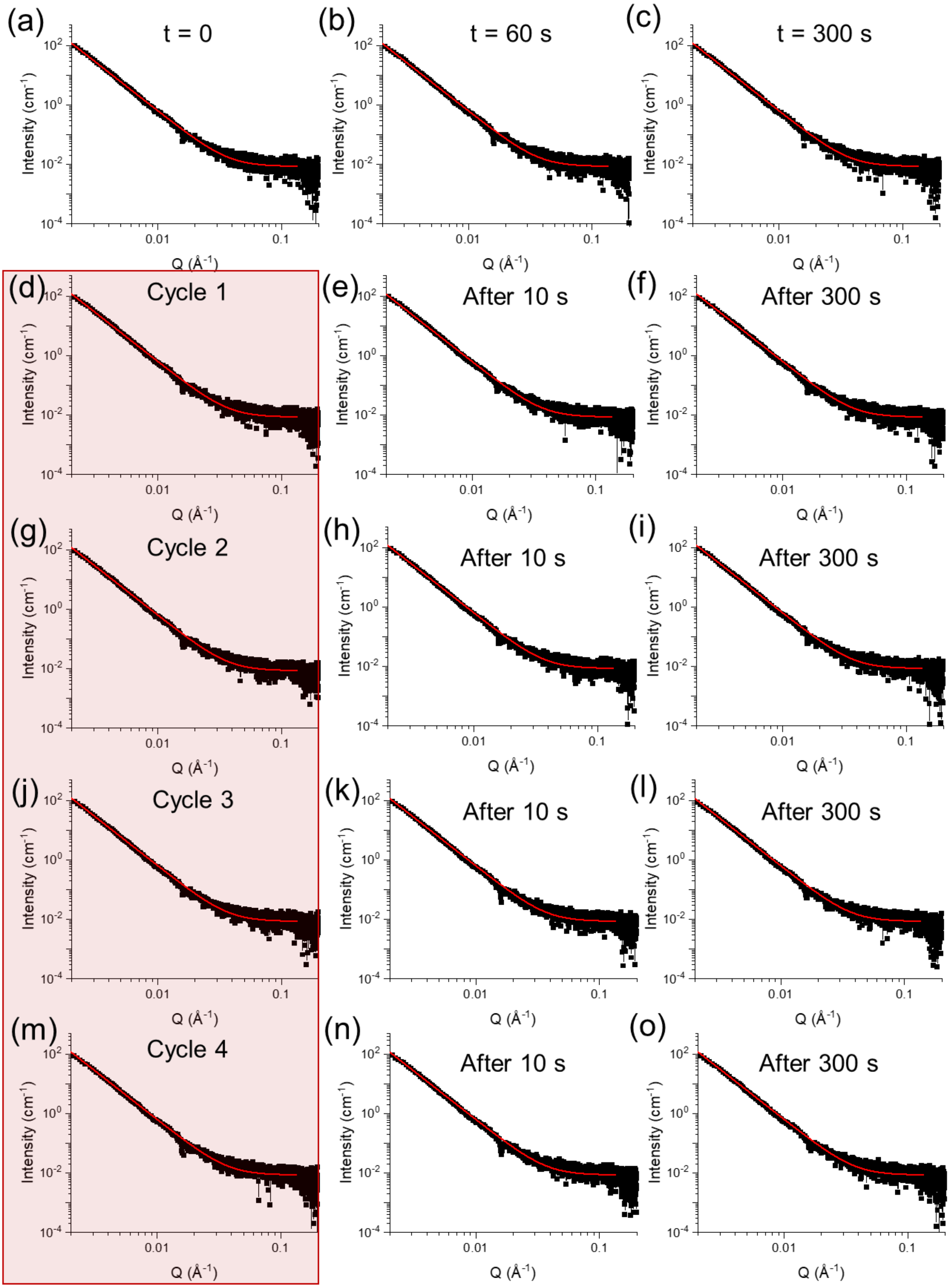

Figure S11. SAXS data (black squares) with model fits (red lines) for a solution of photoacid **1** (1.5 mM) in the dark at pH 7.2 (a) – (c), after 30 seconds of irradiation (d), (g), (j), (m) and after 5 minutes of relaxation (e) - (f), (h) – (i), (n) – (o) over 4 cycles. The red square indicates samples that were measured under irradiation.

Table S3. Fitting parameters for SAXS data in Figure S11. The data was fit to a power law model. Values that were manually added and fixed have been labelled with *.

Photoacid 1	Value	Error
**Scale**	1.92×10^{-7}	4.48×10^{-9}
**Background** / cm^{-1}	0.009*	/
**Power**	3.25	0.004
χ^2	1.06	

2.2.1 5-minute irradiation

To prove no changes could be observed over 5 minutes of irradiation, scattering data was collected for a solution of 1ThNapFF and **1** during irradiation (Figure S12). The scattering patterns all appear to be similar and fit to structural models with the same parameters, highlighting no significant changes over these length scales upon irradiation (Table S4).

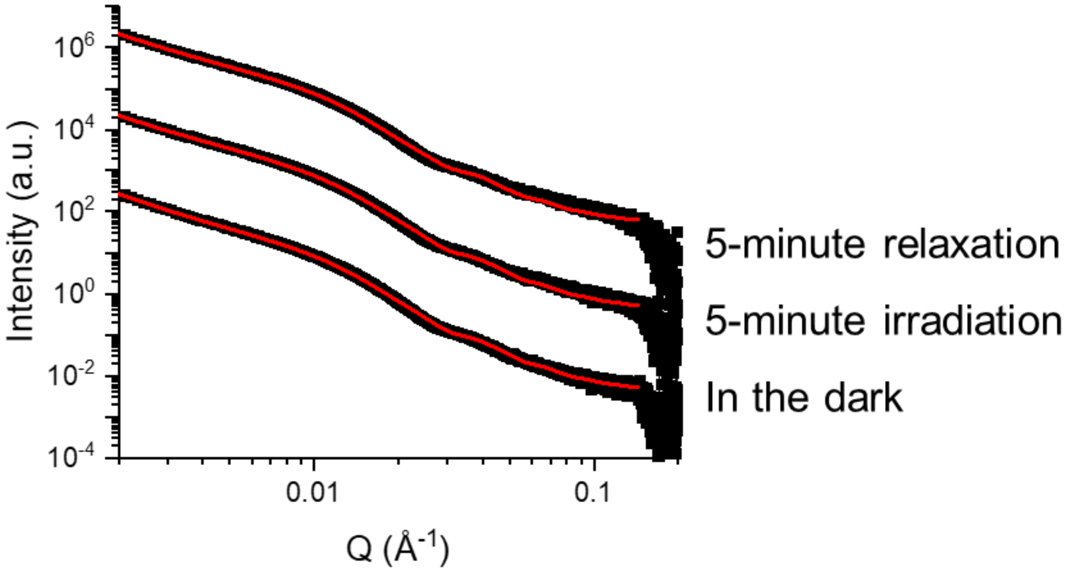

Figure S12. Small angle scattering data (black squares) and model fits (red line) for a solution of 1ThNapFF and **1** (a) in the dark, (b) after 5 minutes of irradiation and (c) 5 minutes of relaxation.

Table S4. Fitting parameters for the small angle scattering data shown in Figure S12. All data was fit to an elliptical cylinder model (A) combined with a power law model (B). Values that were manually added and fixed have been labelled with *.

Sample	In the dark	5-minute irradiation	5-minute relaxation
Background / cm^{-1}	0.004*	0.004*	0.005*
Background error / cm^{-1}	/	/	/
A_scale	$2.56x10^{-4}$	$2.39x10^{-4}$	$2.34x10^{-4}$
A_scale error	$5.94x10^{-7}$	$5.72x10^{-7}$	$5.46x10^{-7}$
A_radius_minor / Å	118.29	118.71	120.62
A_radius_minor error / Å	0.18	0.18	0.20
A_axis_ratio	2.01	2.04	1.93
A_axis_ratio error	0.005	0.005	0.005
A_length / Å	5000*	5000*	5000*
A_length error / Å	/	/	/
B_scale	$4.05x10^{-6}$	$4.95x10^{-6}$	$5.79x10^{-6}$
B_scale error	$6.59x10^{-8}$	$8.75x10^{-8}$	$9.49x10^{-8}$
B_power	2.86	2.78	2.75
B_power error	0.003	0.003	0.003
χ^2	3.95	3.24	2.59

2.3 pH measurements

The pH switching behaviour of a solution of just photoacid (1.5 mM) was tested to observe the behaviour of the solution within the same irradiation times as the composite solution (Figure S13). The solution was irradiated in the same set-up, by switching the light on after 5 minutes in the dark, irradiating the sample for 5 minutes and allowing the system to relax for 5 minutes in the dark. The process is cyclable and reaches a lower pH than the one reached with 1ThNapFF (Figure 2c, main text), suggesting that aggregation or interactions between the dipeptide and **1** might impede the photoacid to fully isomerise under irradiation. In this case, the pH is found to still not fully relax back to the initial 7.2 ± 0.1 over the course the irradiation cycles, indicating that longer times are required to obtain this.

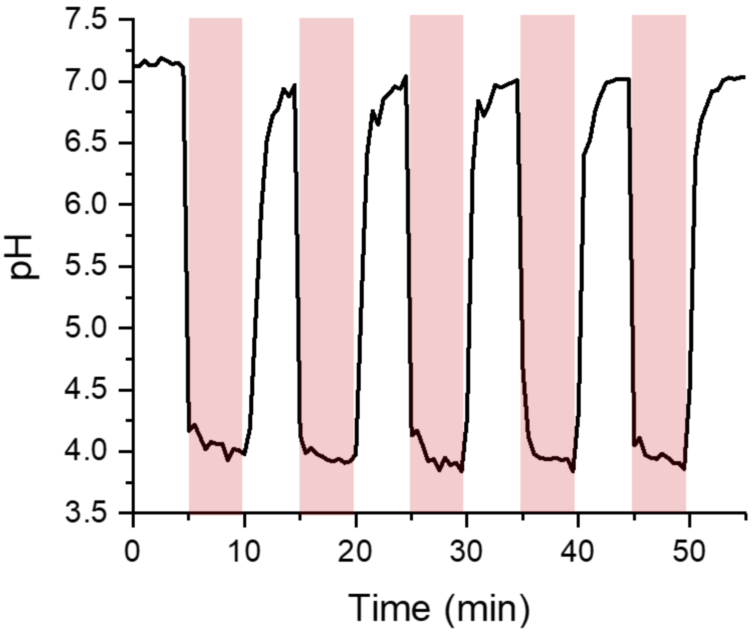

Figure S13. pH measurement of a solution of photoacid **1** (1.5 mM) at pH 7.2 under 5 cycles of 5-minute irradiation with a 450 nm LEDs.

2.4 *In situ* ^{1}H NMR irradiation Measurements

^{1}H NMR spectra of solutions of just 1ThNapFF (1.5 mg/mL), photoacid **1** (1.5 mM) and of the composite solution of 1ThNapFF and **1** (1.5 mg/mL and 1.5 mM) were collected under irradiation. All samples were prepared similarly to the ones in H_2O by using D_2O and 0.1 M KOD. The pD of the samples was adjusted to 7.2 by addition of 1M DCl and 1M KOD.

The changes under irradiation were monitored by ^{1}H NMR experiments (32 scans per spectra). A first spectrum was collected in the dark, followed by 5 minutes of irradiation *in situ*, then 30 minutes of recovery in the dark. Due to the fast changes in the system, it was not possible to use a higher number of scans per spectra.

The data from a solution of just photoacid **1** (1.5 mM) agrees with the reported data by Wimberger *et al.*[23] Due to the low number of scans, the characteristic peak from the spiropyran **2** at 5.80 ppm was too low to distinguish within the noise. However, the disappearance of the characteristic peak of merocyanine **1** at 8.42 ppm can be observed (Figure S14, red square, **1** and Figure S15) under irradiation with 470 nm light.

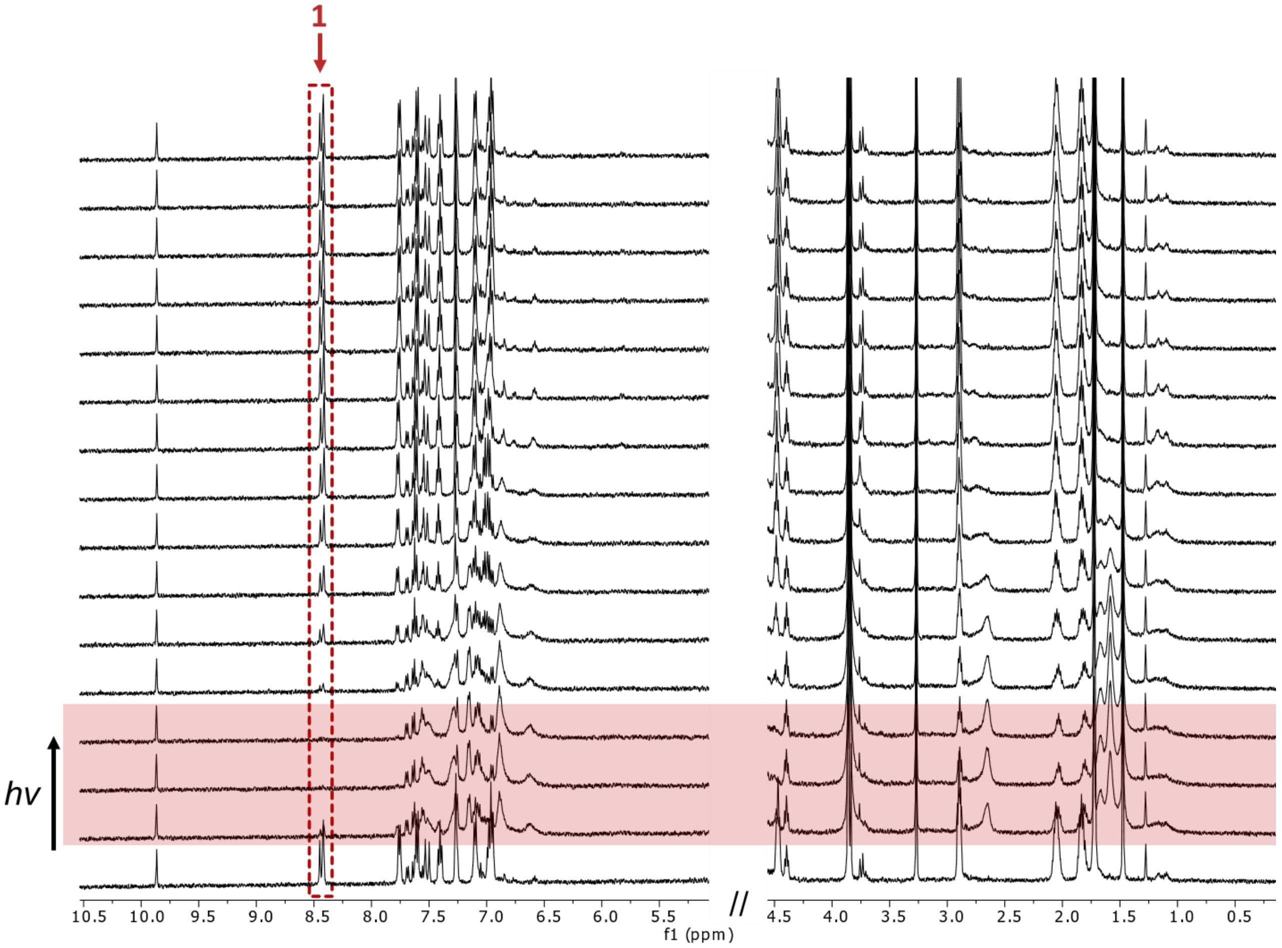

Figure S14. ^{1}H NMR spectra of **1** at pD 7.2 (1.5 mM) under irradiation and relaxation. The bottom spectrum was collected in the dark without irradiation, the three spectra above show the sample under irradiation. The remaining spectra show the sample recovering in the dark. The top spectrum was collected after 30 minutes of relaxation. The red shaded area indicates irradiation. The red dashed square indicates the characteristic peak of **1**.

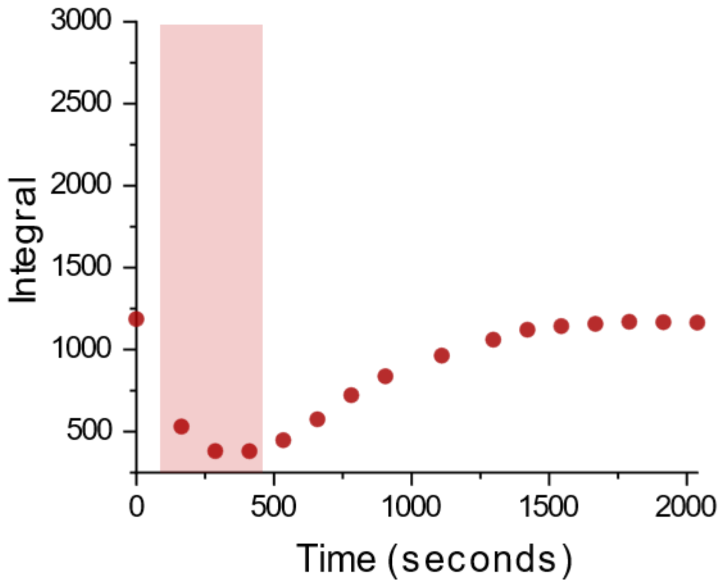

Figure S15. Trace following the absolute integral of the merocyanine characteristic peak at 8.42 ppm under *in situ* irradiation in the NMR at pD 7.2. The data was extracted from the red-dashed rectangle in the ^{1}H NMR spectra in Figure S14. The red-shaded area indicates irradiation.

In presence of 1ThNapFF, the ^{1}H NMR spectra of the solution becomes significantly noisier, with broadening of the peaks related to merocyanine **1** (Figure S16, red shaded area). Due to the partly aggregated state that the dipeptide is found at pD 7.2, NMR peaks related to 1ThNapFF are difficult to observe. However, signal from the dipeptide can be seen as the broad peak at around 7.2 ppm (Figure S16, green shaded area). We hypothesise that interactions between merocyanine form **1** and 1ThNapFF are responsible for the broadening of the peaks. The merocyanine peak and the 1ThNapFF peak both disappear under irradiation (Figures S16, S17). The data indicates that, under irradiation, 1ThNapFF becomes more NMR invisible because of a higher aggregation state of the molecule. This is likely because less interactions with merocyanine **1** can occur under irradiation, favouring more interactions between the dipeptide molecules and leading to an increase in viscosity.

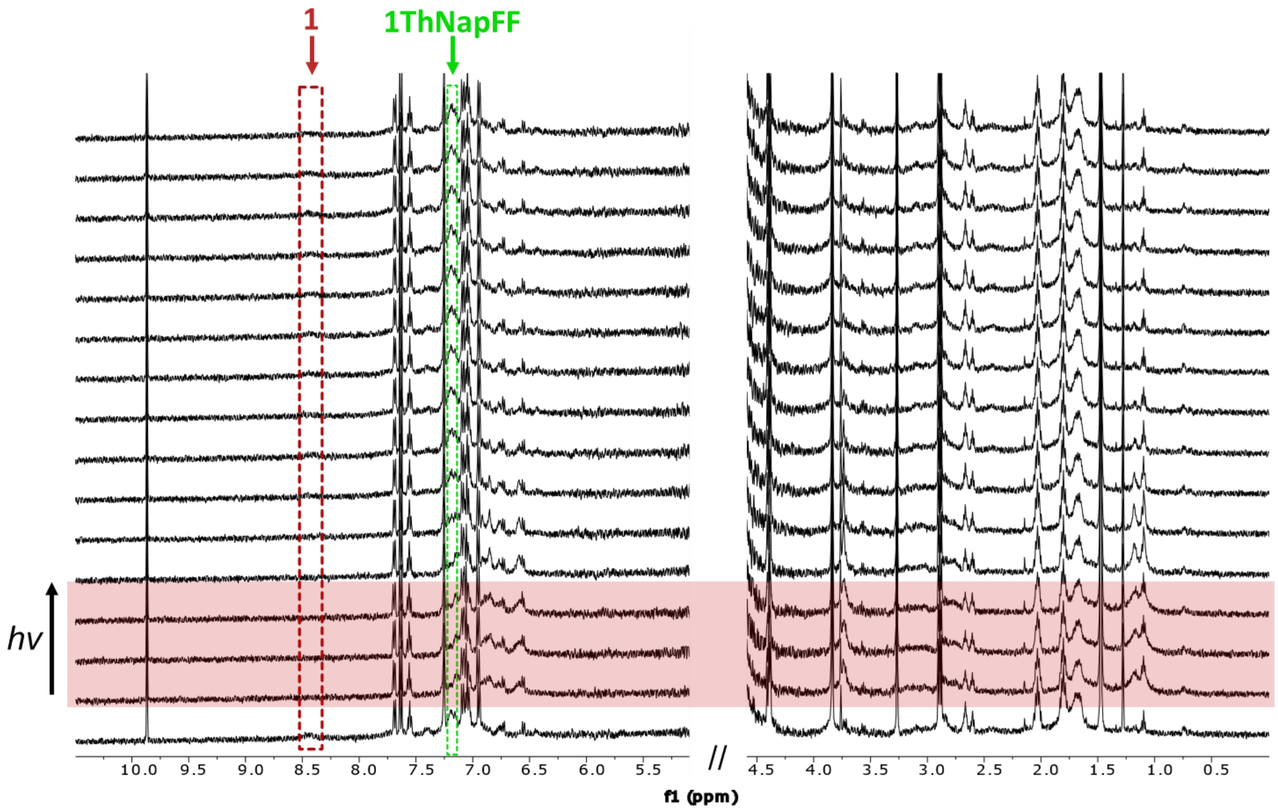

Figure S16. ^{1}H NMR spectra of **1** (1.5 mM) and 1ThNapFF (1.5 mg/mL) at pD 7.2 under irradiation and relaxation. The bottom spectrum was collected in the dark without irradiation, the three spectra above show the NMR spectra of the sample under irradiation. The remaining spectra show the sample recovering in the dark. The top spectrum was collected after 30 minutes of relaxation. The red shaded area indicates irradiation. The red dashed square indicates the characteristic peak of **1**, the green dashed square indicates the 1ThNapFF peak.

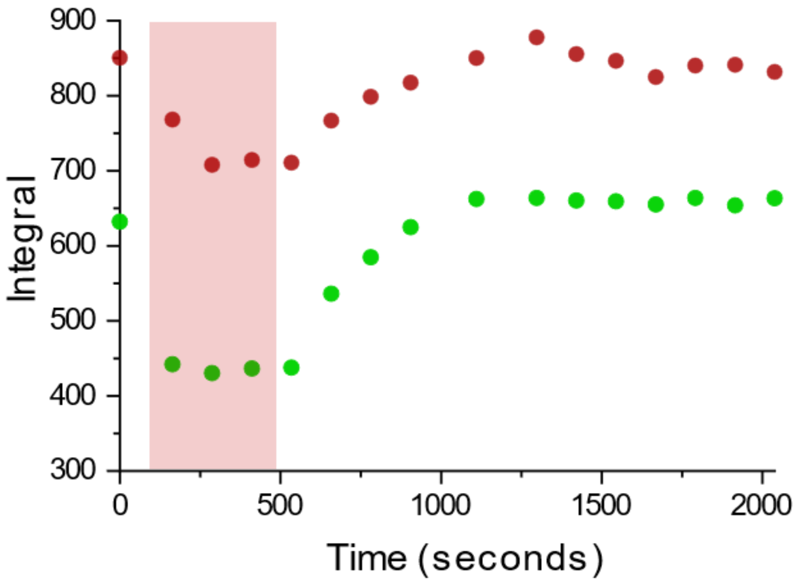

Figure S17. Trace following the absolute integral of the merocyanine characteristic peak at 8.42 ppm and the 1ThNapFF peak at 7.21 ppm under *in situ* irradiation in the NMR at pD 7.2. The data was extracted from the red-dashed rectangle and the green-dashed rectangle in the ^{1}H NMR spectra in Figure S16. The red-shaded area indicates irradiation.

To further prove that the changes are related to the presence of the two interacting species in the system, the spectra of just 1ThNapFF (1.5 mg/mL) was collected under *in situ* irradiation at pD 7.2. As expected, no changes in the spectra can be seen upon irradiation, confirming that the aggregation changes are brought about by the light-induced pH switch in presence of photoacid **1** (Figure S18).

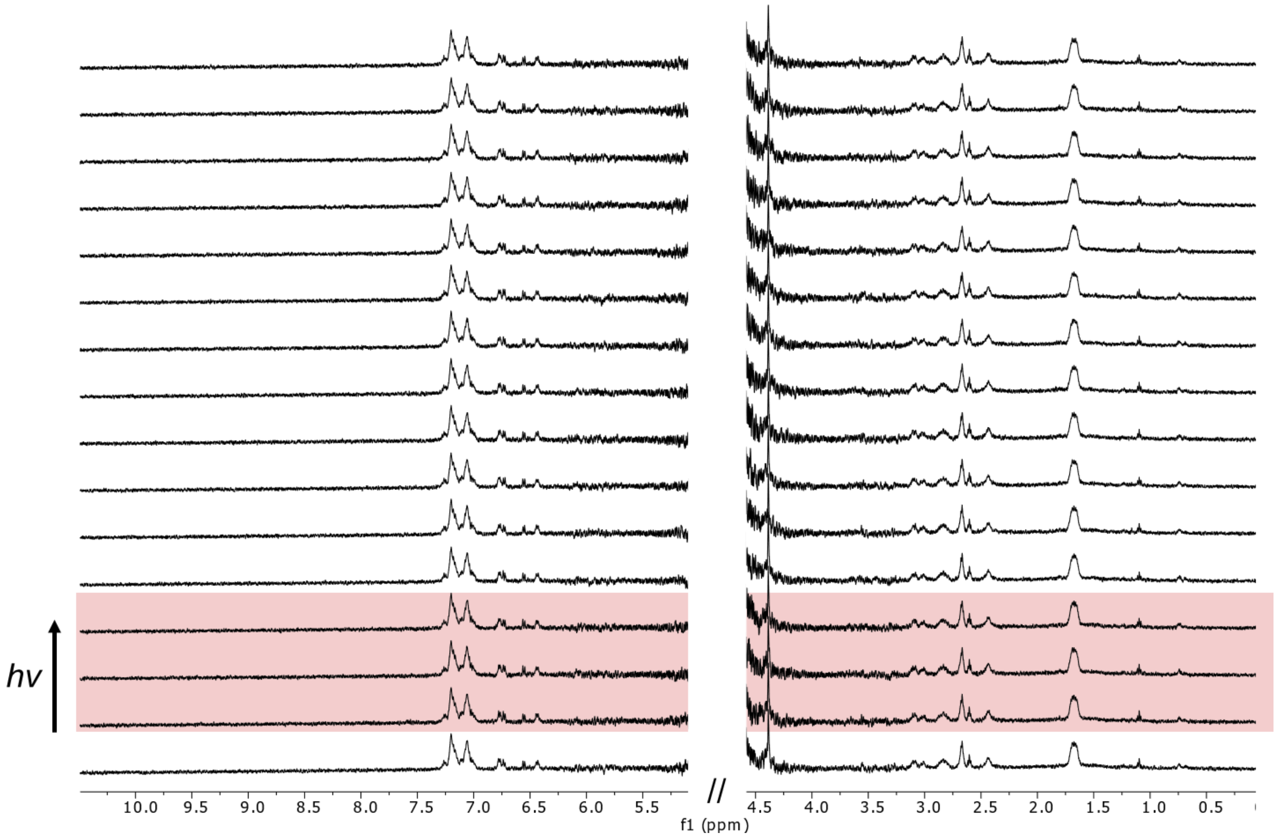

Figure S18. ^{1}H NMR spectra of 1ThNapFF (1.5 mg/mL) at pD 7.2 under irradiation and relaxation. The bottom spectrum was collected in the dark without irradiation, the three spectra above show the NMR spectra of the sample under irradiation. The top spectrum was collected after 30 minutes of relaxation. The remaining spectra show the sample recovering in the dark. The red shaded area indicates irradiation.

2.5 Pressure set-up repeats

To test the viscosifying behaviour of the 1ThNapFF and **1** solution, a custom set-up was used to test changes in pressure upon irradiation (Section 1.6). The data was collected over three cycles using the same solution as in Figure 4b of the main text (Figure S19). We note that the data from the cycle presented in Figure S19a was collected with a lower resolution, resulting in a lower signal-to-noise ratio. As a result, the data was smoothed using Origin with the Adjacent-Averaging method to better compare the datasets (Figure S19, red data). In all cases, there is an increase in detected pressure upon irradiation due to the increase in viscosity.

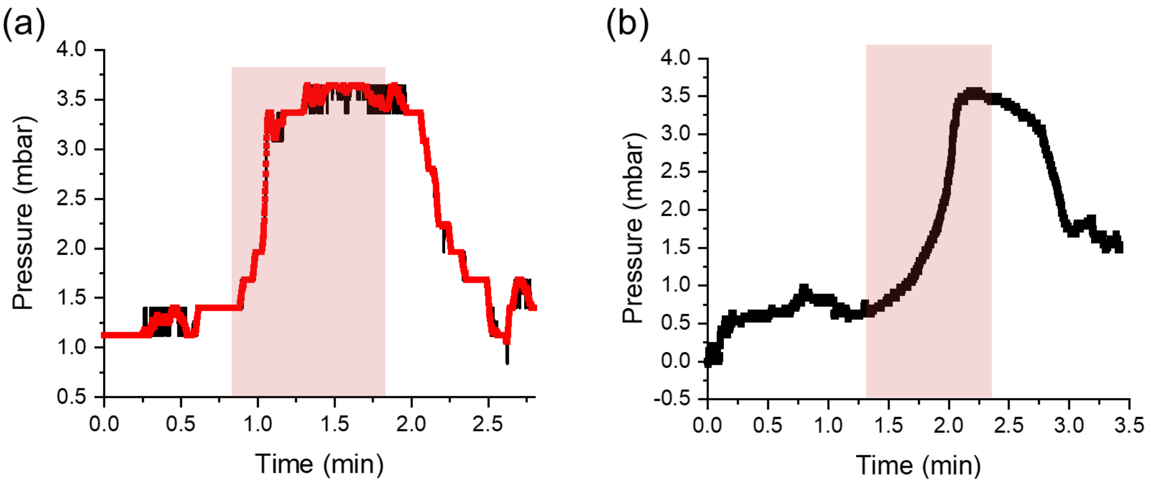

Figure S19. Data collected for a solution of 1ThNapFF (1.5 mg/mL) and **1** (1.5 mM) using the custom set-up to detect pressure over two cycles. The red shaded area indicates irradiation with LED light (λ = 450 nm). In (a), black data is data collected at lower resolution and red data is the smoothed data for ease of comparison.
